# After the first lockdown due to the COVID-19 pandemic: Perceptions, experiences, and effects on well-being in Italian people

**DOI:** 10.3389/fpsyg.2023.1172456

**Published:** 2023-06-02

**Authors:** Venusia Covelli, Elena Camisasca, Gian Mauro Manzoni, Pietro Crescenzo, Alessandra Marelli, Marina Angela Visco, Dario Cafagna, Vincenzo Marsicovetere, Mario Pesce, Manuela Cantoia

**Affiliations:** ^1^Faculty of Psychology, eCampus University, Como, Italy; ^2^Department of Education, Psychology and Communication, University of Bari Aldo Moro, Bari, Italy

**Keywords:** COVID-19 pandemic, psychological effects, Psychological General Well-Being, Perceived Stress, COVID-19-related fear, narratives, mixed-method approach, T-Lab software

## Abstract

**Background:**

Since the COVID-19 pandemic and the subsequent measures of containment, multiple studies have been conducted aimed at assessing the impacts on people’s psychophysical well-being; however, few studies have investigated the general population’s perceptions, experiences, and effects by adopting a mixed-method approach.

**Methods:**

A total of 855 Italian participants completed an online survey, conducted in the period following the first lockdown in Italy. Psychological well-being, perceived stress and COVID-19-related fears were assessed by standardized questionnaires (*Psychological General Well-Being Index-Short version*, *Perceived Stress Scale 10*, and *Multidimensional Assessment of COVID-19-Related Fears*). The process of sense-making of the experience during the lockdown period was also evaluated by means of an open-ended question.

**Results:**

Participants reported a lower level of general well-being, and a higher level of both perceived stress and COVID-19-related fear during the lockdown period compared to the time of the survey (1 month after the resumption of activities). The thematic analysis of responses to the open-ended question revealed two factors and five clusters, which explain the thematic variance among the narratives: the first factor refers to the type of experience (emotional states and feelings vs. objective descriptions of daily activities), while the second concerns positive or negative connotations of the experiences reported.

**Conclusions:**

This study explored the psychological impact of the first lockdown on people’s well-being, and described the process of making sense of the experience during the lockdown 1 month after going back to previous habits. Results highlighted the effectiveness of the mixed-method approach for an in-depth and exhaustive investigation of people’s psychological condition during and after the first lockdown.

## Introduction

Italy was the first Western country to face a COVID-19 outbreak, with over 246,000 documented cases and more than 35,000 deaths due to the disease as of July 26, 2020 (Fortunato et al., 2021). Since the first case of COVID-19 in Italy, detected in a town close to Milan on February 20, 2020, the Italian government employed several drastic measures to contain the epidemic within some areas, to prevent a collapse of the healthcare system. However, these proved unable to contain the run of virus, and on 10^th^ March a national quarantine was adopted for the entire Italian population. The first lockdown concluded on 4th May 2020, followed by a second phase (4th May-15th June 2020), characterized by the possibility of visiting close relations and going out for health and work reasons. After 15th June 2020, the third phase was characterized by a partial return to normality, with the possibility of accessing places of entertainment while retaining the obligation to maintain social distances and to wear a mask in enclosed spaces. Since then, several emergency restrictions have been enacted and subsequently eliminated by the Italian authorities. The restrictive measures primarily limited social behaviors: maintaining a social distance of at least one meter; leaving home only if necessary (foods, pharmacy, medical need); and limiting the number of people gathering in public places. Schools were closed and all educational and didactic activities were provided online. Many hospitals restricted ambulatory services and non-emergency admissions. Work habits were also extensively modified: people were only permitted to go to work if it was not possible to carry out the work remotely.

The restrictive measures impacted on all aspects of daily life and the pandemic caused several fears and worries in many people, especially because the new Coronavirus was previously unknown and spread very quickly, claiming hundreds of thousands of victims in a very short time (Lanciano et al., 2020). All these factors contributed to a great distress worldwide (Yu et al., 2020), forcing people to develop new coping strategies to manage daily life, with relevant implications for mental health (Gritti, 2020; Gritti et al., 2020; Li et al., 2020; Mari et al., 2020; Mariani et al., 2020; Yao et al., 2020).

Several studies highlighted a consistent variety of psychopathological manifestations of self-isolation, quarantine, and inactivity (Brooks et al., 2020; D’Ambrosi et al., 2020; Pfefferbaum and North, 2020). Principal outcomes are related to increased levels of stress, anxiety, emotional instability, worry, and depressive symptoms (Winkler et al., 2020), matching the most commonly reported effects in populations that have suffered similar conditions in past adverse events (Ettman et al., 2020). In the Italian scenario, Casagrande et al. (2020) found that the principal predictors of psychological lockdown-related distress were linked to time (long duration of quarantine, boredom), fear of infection (to be infected or infect a close person), agency (frustration), communication (inadequate or ambiguous information), economic (financial loss, impossibility to work), and stigma. Moreover, several surveys were administered to evaluate the level of distress. Mazza et al. (2020) found that gender played a role in the development of anxiety and depression as well as increased levels of stress, while age was a significant factor only for anxiety levels and perceived stress.

During the pandemic, high levels of distress, anxiety, depressive symptoms, negative well-being, decreased quality of sleeping, and decreased vitality and perceived general health are examples of the disturbances experienced due to confinement and uncertainty (Casagrande et al., 2020; Iorio et al., 2020; Moccia et al., 2020; Orgilés et al., 2020; Parrello et al., 2021; Sommantico et al., 2021). Mazza et al. (2020) highlighted that a history of stressful situations and medical problems increased anxiety and depression during national lockdowns. Other authors (Biondi and Iannitelli, 2020; D’Ambrosi et al., 2020; Femia et al., 2020; Boursier et al., 2021) also reported emotional states of anxiety and sadness. This increasing psychological discomfort was evident from the first weeks of the lockdown. To address and prevent it, the Italian National Council of Psychologists encouraged registered specialists to provide online psychological support to the population (Crescenzo et al., 2021a,b; Limone and Toto, 2022).

The COVID-19 pandemic and the subsequent measures of containment have stimulated multiple studies aimed at assessing the impact both on medical staff (Simione and Gnagnarella, 2020; Chirico et al., 2021; Saladino et al., 2022; Tarchi et al., 2022) and on people’s psychophysical well-being (Saladino et al., 2020).

During the last couple of years, many studies probed the experience of the first wave of the pandemic through a mixed-method design, collecting both qualitative and quantitative data. Among those studies, those which combined validated questionnaires and open-ended questions or in-depth interviews focused on specific populations, mainly health care workers, nurses and doctors (e.g., Svantesson et al., 2022; Harris et al., 2023; Richards et al., 2023), but also medical students (Rolland, 2022), family caregivers (Leleszi-Tróbert et al., 2022), women with young children (Wandschneider et al., 2022), children with obesity (Welling et al., 2022), supermarket workers (Mayer et al., 2022), and university students (Mourad et al., 2022). To our knowledge, three studies explored the effects of stress during the first wave of the pandemic through a mixed-method design. Two studies focused on the adult population: Bin Helayel et al. (2022) examined stress-related disorders during the quarantine period to underscore the importance of both an early detection of the symptoms and of supporting quarantined individuals. Cipolletta et al. (2022) focused on the episode of the worst experience according to the levels of peritraumatic distress. A third study investigated the impact of the pandemic on youth’s daily life according to their levels of post-traumatic growth, their sociodemographic characteristics, and their coping strategies, mainly maintaining social contacts, engaging in leisure activities, and physical exercise (Hébert et al., 2022).

Assuming a different perspective, our research interest was on the impact of the first lockdown on people’s well-being, and our study aimed to explore how participants made sense of their experience of the first lockdown 1 month after the resumption of activities (after the third phase). For exploratory and descriptive purposes, we asked people to think back to the period of the first lockdown and to describe how they experienced that period, by means of an open-ended question. In addition, we asked them to rate their state of health and quality of life and sleep during the first lockdown compared to the moment of their participation in the survey. Finally, we measured their state of well-being, perceived stress, and perceived risk of contracting the virus at the time of survey participation through validated questionnaires.

Our research questions concerned:

- **RQ1**: The extent of the psychological impact of the first lockdown, 1 month after going back to one’s own previous habits: were participants able to differentiate between the experience of lockdown compared to their actual status as assessed through both self-evaluation (state of health and quality of life and sleep) and validated questionnaires (well-being, perceived stress, and perceived risk of contagion)?
- **RQ2**: The process of sense-making of one’s experience during the lockdown: we wanted to assess if participants still felt the fear of the pandemic, the fear of contagion, and the difficulty in resuming activities after the first lockdown or were already able to recall those days through more objective frames. We were also interested in the comparison between the answer to the open-ended question and the results of the questionnaires that measured the level of stress, fear of contagion, and well-being.

Based on the research questions, we hypothesized that participants would report: (1) a more positive self-evaluation of quality of life, sleep, and well-being levels at the time of the survey (1 month after the resumption of activities) compared to the lockdown period; and (2) a worse self-evaluation of perceived stress, perceived risk of contagion, and COVID-19-related fears during the lockdown compared to the time of the survey. We also hypothesized a consistency between the content reported in the open-ended question and the results of the questionnaires measuring stress level, fear of contagion, and well-being.

## Materials and methods

### Study design and data collection

In this descriptive and explanatory mixed-method study, we collected both quantitative and qualitative data to provide an integrated view of how the COVID-19 pandemic impacted people’s psychophysical well-being (McAdams et al., 2001; Anguera et al., 2020; Cipolletta et al., 2022; Mourad et al., 2022). We collected and analyzed self-report validated questionnaires measuring psychological well-being, as well as qualitative data through an open-ended question about the lived experience during the first lockdown period.

A total of 1,325 adults volunteered for the survey and gave their informed consent to data treatment. After eliminating people that answered the first 14 questions only (sociodemographic section), the final sample comprised 855 adults (68.4% females) aged between 18 and 70 years (*M* = 40.8; SD = 13.22). Data were collected using an internet-based self-administered survey delivered in Italian by Survey-Monkey, from the 1^st^ August till the 15^th^ August 2020. The survey was spread via email and social media (LinkedIn, Facebook, and WhatsApp). The online questionnaire required an estimated 15–25 min for the participant to complete it. Participants received information about the aim and procedures of the survey and were asked for informed consent before starting the survey. People over 18 years of age who residing in Italy during the COVID-19 outbreak were eligible to respond to the survey. Participation was completely voluntary, and participants could withdraw from the study at any time.

To assess COVID-19’s psychological influence on psychological well-being, we instructed participants to respond to the questionnaire with reference to the COVID-19 epidemic. Data collected when a participant had either taken less than 15 min to complete the test or had not addressed one or more questions were disregarded to ensure the quality of the study.

### Measures

#### Sociodemographic and COVID-19 section

All participants were asked to provide socio-demographic information (e.g., gender, age, level of education, marital status, region of residence, number of children, profession, and changes in work conditions). In addition, they answered some questions regarding their experience during the COVID-19 pandemic in a dedicated section created *ad hoc*. Information was collected about exposure to COVID-19, family members infected by COVID-19, or loss of a family member due to the virus. Participants were asked whether their health condition was aggravated by the virus and about their perceived risk of contracting the virus; they also assessed their perceived quality of life, health status, and sleep quality during the first lockdown and at the time of the survey. Finally, participants described via an open-ended question how their experiences characterized their life during the first lockdown: “*Think back to the lock-down period and tell us about yourself in that period: you can start wherever you want and say whatever you want, you can talk about it or focus on the episode that best represents what you experienced*.”

#### Psychological general well-being index-short version (PGWI-S)

This questionnaire (Grossi et al., 2006) provides a general evaluation of self-perceived psychological health and well-being over the past 4 weeks. It consists of six items that evaluate different aspects of psychological well-being (Anxiety, Vitality, Depressed mood, Self-control, Positive well-being, and Vitality). Each item is rated on a 6-point Likert scale from 0 to 5 (maximum global score = 30). Responses are scored so that higher total PGWB-S scores indicate higher psychological well-being. Cronbach’s alpha for the PGWI-S is 0.89.

#### Perceived stress scale 10 (PSS-10)

This is a 10-item questionnaire that measures the degree of perceived stress over the previous month (Cohen et al., 1983; Fossati, 2010). Participants indicate how often they have felt or thought in a certain way, also including their levels of experienced stress. All the items can be answered on a 5-point Likert scale from 0 (never) to 4 (very often). Higher scores indicate higher stress levels. This is a scale that has reported a good internal and structural consistency. Cronbach’s alpha for the PSS is 0.80.

#### Multidimensional assessment of COVID-19-related fears (MAC-RF)

This questionnaire (Schimmenti et al., 2020) consists of eight items that investigate eight types of COVID-19-related fears: fear of the body, fear for the body, fear of others, fear for others, fear of knowing, fear of not knowing, fear of action, and fear of inaction. The items are grouped into four subscales: fears related to the body, fears related to meaningful relationships, difficulties in cognitive monitoring of concerns, and behavioral difficulties related to fear. Respondents rate all eight items on a 5-point Likert scale ranging from 0 (very unlike me) to 4 (very like me). In literature, authors reported a single-factor structure, satisfactory reliability whereas convergent validity was based on its positive correlation with overall psychopathology. Cronbach’s alpha for the MAC-RF is 0.84.

### Data analysis

Descriptive statistics were computed to describe the socio-demographic characteristics of the sample. The Welch *t* test when variances were not homogeneous or the Mann–Whitney test when distributions were not normal were used to assess differences between two groups (e.g., gender), while ANOVA, for correction of non-homogeneous variances, or the Kruskal Wallis test for non-normal distributions were used to assess differences between more than two groups (e.g., education level and civil status). The Wilcoxon rank sum test was used to evaluate changes in quality of life and sleep, health perception, and risk of contracting COVID-19 between the retrospective first lockdown and the survey period (1 month after). Biserial correlations were used to assess changes between main measures and socio-demographic characteristics. Finally, path analysis was used to assess if: (1) psychological well-being was predicted by COVID-19-related fears both directly and through the mediation of perceived stress; and (2) gender moderated the mediation effect, i.e., the effect was stronger for females than males. Data analyses were performed through the software SPSS and JAMOVI (version 2.3.21).

To analyze the answers to the open question in depth and to detect people’s experience during the lockdown, a lexicographic analysis was performed with an automatic text analysis tool, the T-LAB software (version T-Lab Plus 2020, Lancia, 2004). The procedure of linguistic and statistical analyses on collected texts consisted of several stages: (1) automatic data processing stage (normalization, vocabulary construction, and corpus segmentation); (2) keywords selection; (3) thematic analysis of elementary contexts; and (4) processing of the results prior to interpretation. Following this process, the results were interpreted by multiple judges (VC, PC, and EC). As for the third phase, the software performed a thematic analysis of the elementary contexts, i.e., a cluster analysis with a bisecting K-Means algorithm, excluding all those elementary contexts that do not present at least two co-occurrences. This procedure constructed a mapping of the corpus contents according to the co-occurrence of the selected keywords and it allowed to organize the text in thematic clusters and analyze recurring words and semantic expressions. That is, clusters were characterized by sets of lexical units that shared the same elementary context units. To choose the number of clusters that were considered for interpretation, reference was made to the number of factors organizing them in space.

## Results

Participants’ sociodemographic characteristics are reported in Table 1. Most participants (65.3%) lived in the North-Center of Italy and had a high level of education (71.3% having completed a university degree or a post-university degree; 52% having completed high school). Most (73%) were employed (12.5% unemployed or retired and 11.8% students). Among workers (73% of respondents), 27% reported that they never stopped working (even during the lockdown period), 15% went back to work after the first lockdown, and 4.1% did not work or lost their job because of the lockdown.

**Table 1:** Socio-demographic characteristics (*N* = 855).

	N (%)
*Gender*	
Male	270 (31.6)
Female	585 (68.4)
*Education*	
Primary school	3 (0.4)
Secondary school	15 (1.8)
High school	445 (52)
Degree	147 (17.2)
Master’s degree/higher	155 (18.1)
Master	90 (10.5)
*Marital status*	
Single	336 (39.4)
Married	326 (38.1)
Cohabitant	109 (12.7)
Divorced	71 (8.3)
Widow	13 (1.5)
*Region of residence*	
Northwest	272 (31.8)
Northeast	115 (13.4)
Center	172 (20.1)
South	169 (19.8)
The Islands	127 (14.9)
*Work conditions*	
Manager, entrepreneur, freelancer	181 (21.2)
Employee, self-employed	365 (42.7)
Laborer	50 (5.8)
Armed forces	28 (3.3)
Not employed	71 (8.3)
Retired to work	36 (4.2)
Housewife	23 (2.7)
Student	101 (11.8)
*About your job (73% of sample)*	
I resumed going to work, after having stopped working during the lock down	128 (15)
I went back to work, after having worked in smart-working from home during the lock-down	116 (13.6)
I have never stopped working, even during the lock-down	231 (27)
I am always working in smart working	110 (12.9)
I am not working; I lost my job because of the lock-down	35 (4)
I am not working and was not working even before the lock-down	120 (14)
Other	115 (13.5)

Most participants (97.9%) declared they were not affected by COVID-19, while 1.5% spent a period at home in quarantine and 0.6% declared they spent a period in the hospital because of COVID-19. Most (75.8%) also declared they did not have a health condition aggravated by COVID-19, while a minority (17.7%) reported that the coronavirus brought them to difficult health conditions, both physical and psychological. The majority (90.9%) also declared not to have suffered from the loss of relatives and friends because of COVID-19 and 69.1% did not contaminate their family. Finally, almost half the participants (41.3%) declared that COVID-19 caused financial problems for their families.

### Daily life, health status, perception of risk, COVID-19-related fears, perceived stress, and psychological well-being

Some participants reported that the COVID-19 pandemic and the first lockdown impacted their daily life habits significantly: 26.9% had no social contact (they could not visit their loved ones such as parents, partners, or friends), 20.3% were not able to go out freely (even for a walk), and 10.6% stayed indoors all the time (see Table 2). Health perception, quality of life, and sleep improved, and the perceived risk of coronavirus infection reduced, from their retrospective assessment of the lockdown period to the survey time (see Table 3).

**Table 2:** In which aspect of your life has the Coronavirus had the greatest impact? (multiple choice).

	*N*	% (of cases)
Social contact: I could not visit my loved ones (parents, partner, friends, etc.).	587	26,90% (69,70)
Being able to go out freely, even for a walk	442	20,30% (52,50)
Environmental insulation (staying indoors all the time)	231	10,60% (27,40)
The ability to engage in recreational activities (cinema, theater, shopping, aperitifs, dinners at restaurants, etc.).	216	9,90% (25,70)
Being able to engage in sports activities	182	8,40% (21,60)
Occupation: having to work/study from home	144	6,60% (17,10)
Employment: I lost my job/my income has decreased	111	5,10% (13,20)
Family management: taking care of the family without external help/services (e.g., managing children or household chores)	96	4,40% (11,40)
Taking care of myself (physical activity, hairdresser, beautician, SPA, massage, etc.)	83	3,80% (9,90)
My health status or that of a family member (I was/They were ill, but I/they have recovered)	49	2,20% (5,80)
Loved ones (I lost someone dear to me to the Coronavirus)	38	1,70% (4,50)
Total	2,179	100%

**Table 3:** Retrospective changes in quality of life and sleep, health perception, and risk of contracting COVID-19 between the first lockdown and 1 month after its conclusion.

Misure	During the first lockdown M (SD)	1 month after the resumption of activities M (SD)	*Z* statistic
Quality of life	3.29 (0.978)	3.78 (0.784)	−13.826***
Health condition	3.56 (1.065)	3.75 (1.004)	−7.261***
Quality of sleep	3.29 (1.065)	3.78 (1.004)	−7.261***
Risk of contracting COVID-19	2.24 (1.393)	2.10 (1.248)	−2.688**

SD: standard deviation.

Wilcoxon rank sum test, non-parametric, (value of *p*) does not assume normality. Significance values reported as *p < 0.05; **p < 0.01; ***p < 0.001.

About COVID-19-related fears, mean and median MAC-RF total scores (21 and 21.5 respectively) resulted to be above the cut-off (20) that, according to Schimmenti et al. (2020), indicates the initial presence of pathological fears in potential need of a clinical consultation. Indeed, most participants (57.9%, *n* = 468) had a score equal to or higher than 20 and 31.1% (*n* = 251) had a score equal or higher than 12, the first threshold indicating the presence of psychological problems in mental, relational, and behavioral functioning. Women showed a significantly but slightly higher median MAC-RF total score than men (Mann–Whitney *U* = 57,745, *p* < 0.001, Rank Biserial Correlation = 0,174) and a higher percentage of women than men reported a score equal to or above 20. With respect to education level, participants with a Ph.D./Master/Specialization showed a lower mean score (19.3), and a statistically significant but small difference was found only between them and those with a High School Diploma (21,5). With respect to Civil Status, unmarried participants showed a higher mean score (22.1), and statistically significant differences were found between them and both those cohabiting and those separate/divorced (19.8 and 18.8 respectively).

No statistically significant difference was found in any of the following comparisons: (1) participants who declared to have been infected (*n* = 15) or not (*n* = 803); (2) participants who declared to have loved ones infected (*n* = 254) or not (*n* = 554); and (3) participants who declared to have lost someone due to COVID-19 (*n* = 75) or not (*n* = 733).

Statistically significant and negative correlations were found between the MAC-RF total scores and quality of life, health satisfaction, sleep quality (the higher the values, the lower the score), and risk perception of coronavirus infection (the lower the risk, the lower the score) and the survey period.

With respect to perceived stress, mean and median PSS total scores (19.56 and 20 respectively) indicate moderate stress (Cohen et al., 1983; Lee, 2012). Women showed a significantly but slightly higher median total score than men (Mann–Whitney *U* = 58,469, *p* < 0.001, Rank Biserial Correlation = 0.165). Statistically significant and negative correlations were found with quality of life, health satisfaction, sleep quality (the higher the values, the lower the score), and risk perception of coronavirus infection (the lower the risk, the lower the score).

Psychological well-being (PGWBI-S) mean and median total scores (62.13 and 62.33 respectively) were below the cut-off (70.87) and the differences were statistically significant (one sample *t* = −15.854, df = 809, *p* < 0.001, Cohen’s d = 0.557; one sample Wilcoxon *W* = 77.169, *p* < 0.001, Rank Biserial Correlation = 0.53). Women showed a significantly but slightly lower median total score than men (Mann–Whitney *U* = 58,469, *p* < 0.001, Rank Biserial Correlation = 0.181). With respect to education level, civil status, and the other factors, no statistically significant difference was found. Statistically significant and positive correlations were found with quality of life, health satisfaction, and sleep quality (the higher the values, the higher the score).

**Figure 1:**
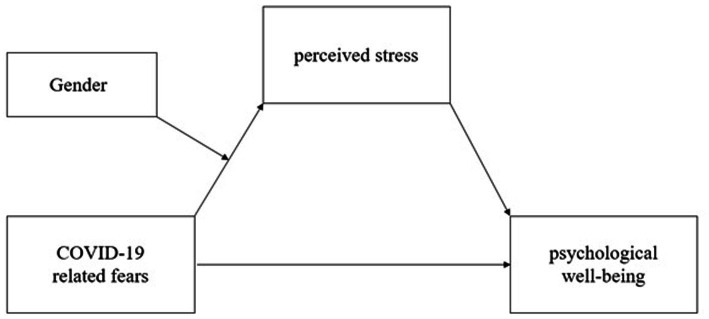
The mediation model.

Finally, path analysis showed that the predictive effect of COVID-19-related fears on psychological well-being was both direct and mediated by perceived stress, and that gender moderated the predictive effect of COVID-19-related fears on perceived stress but did not moderate the predictive effect of perceived stress on psychological well-being (see Figure 1).

### Lockdown experiences: A qualitative insight

**Figure 2:**
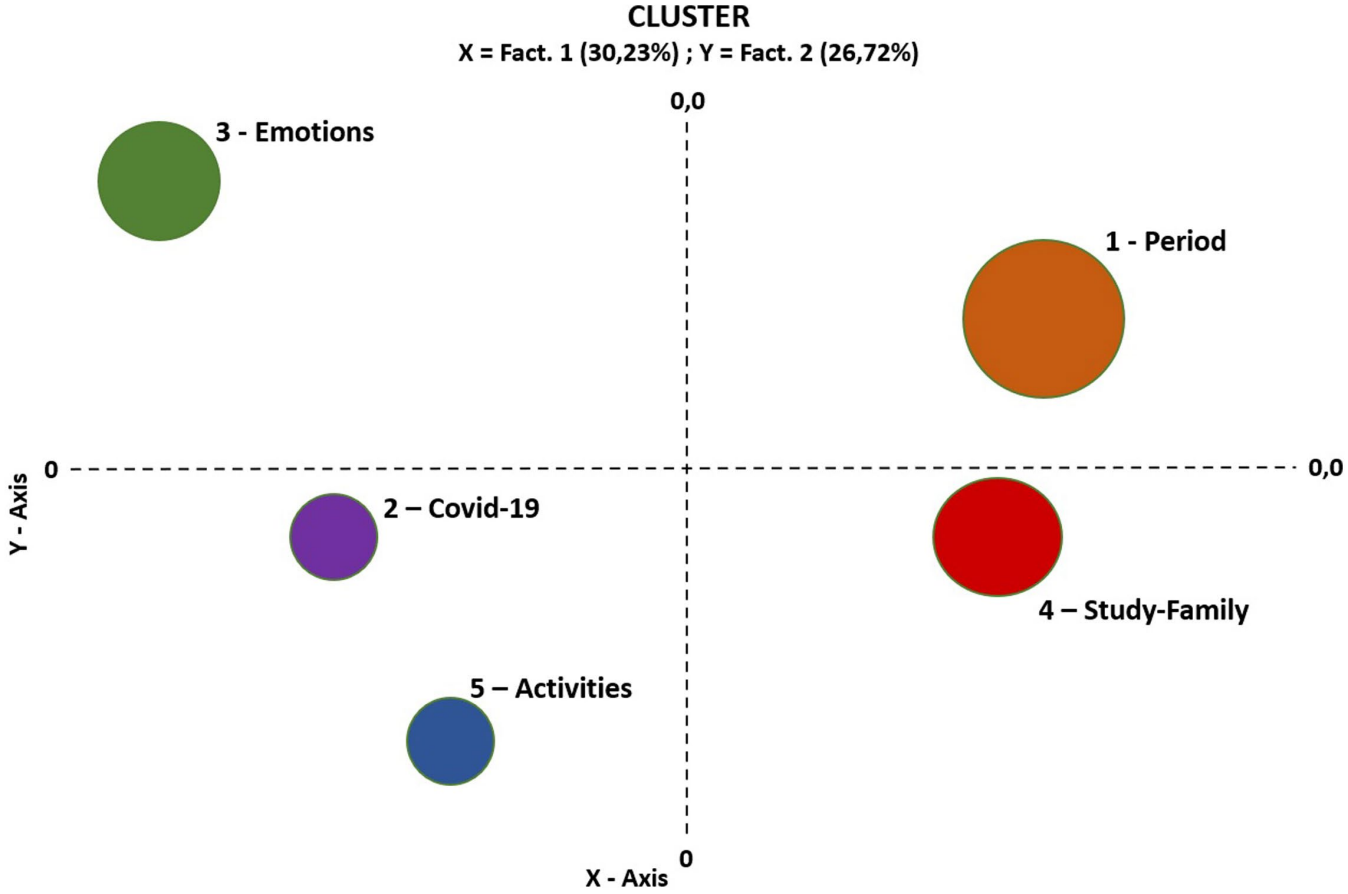
Elementary contexts representation.

The thematic analysis revealed two main factors (see Figure 2) explaining the thematic variance among the qualitative responses. The first vertical axis explains 30.23% of the lexical variance and relates to the type of the experience reported. This first axis contrasts narratives related to emotional states, experiences, feelings (up to the continuum, mainly characterized by terms such as “anxiety,” “affection,” “thoughts,” “nightmare,” “fear,” and “loneliness”) to narratives related to descriptions of activities or everyday life from an objective point of view (down to the continuum, mainly characterized by words such as “working,” “smart working,” “quitting,” “continuing,” “from home,” “lay-offs,” and “exam”). This axis articulates clusters 3 and 1 up to the continuum, and cluster 2, 4, and 5 down to the continuum. The second horizontal axis explains 26.72% of the lexical variance and mainly concerns the positive or negative connotation of the experience analyzed. This axis opposes, at the positive pole, elementary context unit related to a positive experience lived by respondents (i.e., words such as “freedom,” “serene,” “reflection,” “opportunity,” and “strong”), and at the negative pole, elementary context unit related to a negative experience lived by respondents (i.e., related words such as “distress,” “fear,” “sadness,” “loneliness,” “distance,” and “anger”). This axis also opposes clusters 1 and 4 on the right of the continuum, and clusters 3, 2, and 5 on the left of the continuum (Table 4).

**Table 4:** Cluster of lockdown experiences.

Cluster	*N*	%	Name
1	336	27.2	Period
2	197	16.12	Covid-19
3	237	19.39	Emotions
4	271	22.18	Study-family
5	181	14.81	Activities

Let us now describe each cluster specifically (Table 4). Cluster 1, labeled “period,” which explains 27.2% of the thematic variance, classifies 336 elementary contexts (out of the 1,272 classified in the whole corpus). This cluster relates mainly to the qualification of the period either as positive (“*It was fruitful, important*”), or negative (i.e., words such as worry, fragility, stress, withdrawal. For example: “*It has been a beneficial period as I finished my online third year of motor science studies and also preparing my final thesis. This period reunited the family, changed the habits of free time spent playing board games, making special recipes and exercising in the home gym. In summary, it was a period of reflection and hope*”). Cluster 2 labeled “COVID-19,” which explains 16.12% of the thematic variance, classifies 197 elementary contexts (out of the 1,272 classified in the whole corpus).

This cluster relates mainly to the impact of COVID-19 on the physical dimension (such as loss of taste and sense of smell), tampons, daily hospitalization, disease. For example: “*So my husband manages to go out to look after them [children] but I do not, I’m just at home. After a few days when I spray some perfume around the house… I do not smell anything… I do not smell anything… I no longer smell anything… as in a chain everyone follows me, my husband and then Laura… for a good twelve days, no more smells…”* Cluster 3 labeled “Emotions,” which explains 19.39% of the thematic variance, classifies 237 elementary contexts (out of the 1,272 classified in the whole corpus). This cluster relates mainly to emotional states related to the lockdown experience. For example: “*A lot of fear at the beginning of having to stay indoors, fear of getting sick but especially of my loved ones getting sick… then a feeling of safety in staying indoors and fear of going out once the lockdown was over.”* Cluster 4 labeled “Study-family,” which explains 22.18% of the thematic variance, classifies 271 elementary contexts (out of the 1,272 classified in the whole corpus). This cluster concerns how people tried to reconcile different activities (description of difficulties at school, at university, in the family, etc.). For example: “*I am a working student so after work I spent a lot of time studying and playing with my daughter and family. We dusted off board games and did various activities. I helped my daughter with distance learning.”* Cluster 5 labeled “Activities,” which explains 14.81% of the thematic variance, classifies 181 elementary contexts (out of the 1,272 classified in the whole corpus). This cluster mainly relates to work activities (in presence vs. smart working or layoff). For example: “*We asked the company if it was possible to continue with smart working in view of the emergency. The company agreed for the first week but the second I would have to return, but by luck the Prime Minister obliged the companies to work remotely until the situation would return to normal.”*

## Discussion

Unprecedented situations and fears deeply affected the global population during the first lockdown that was imposed to limit the SARS-COV-2 spread (Yu et al., 2020). The psychological extent of this experience over time was not predictable. Increased levels of stress, anxiety, emotional instability, worry, depressive symptoms, negative well-being, decreased quality of sleeping, decreased vitality and perceived general health, were reported in several studies across countries (*inter alia*, Brooks et al., 2020; Casagrande et al., 2020; D’Ambrosi et al., 2020; Moccia et al., 2020; Orgilés et al., 2020; Winkler et al., 2020; Sommantico et al., 2021). This mixed-method online survey had exploratory purposes and was aimed to detect the psychological impact of the first lockdown on people’s well-being, and to understand the process of making sense of the experience during the lockdown, 1 month after going back to previous habits.

Our first research question dealt with the extent of the psychological impact of the first lockdown, 1 month after going back to previous habits (after the third phase). We asked participants to evaluate the experience of lockdown and their actual status and we hypothesized that the participants would report a more positive self-evaluation of quality of life, sleep, and well-being levels at the time of the survey (1 month after the resumption of activities) compared to the lockdown period and a worse self-evaluation of perceived stress, perceived risk of contagion, and COVID-19-related fears during the lockdown compared to the time of the survey.

By the end of the first lockdown respondents began to feel an improvement in perceived quality of life, quality of sleeping, and satisfaction about health. At the moment of the survey, participants acknowledged that their situation was still improving, they appreciated a better quality of life and slept better, they also were more confident in their health and felt more protected from the risk of contagion. Instead, during the lockdown, fear of Covid 19 still reached quite critical and pathological levels; in particular less educated, unmarried, and younger respondents were more impacted. According to Doshi et al. (2021) the higher the education level, the higher the ability to use information on the risk of contracting the infection, and the lower the possibility to develop related fears. The condition of being younger or unmarried may be related to a higher feeling of solitude (Rosenberg et al., 2021; Banerjee and Kohli, 2022). Overall, these data are coherent with the statement given by participants that social and physical withdrawal were retrospectively the most critical aspects of the first lockdown: fear was felt more strongly by people who lived alone or had fewer strategies, either cultural or personal ones, to deal with it. Fear was also stronger in women: as already known in the literature, women and men reported quite different experiences of the first lockdown, with women generally more psychologically affected by that period (Hossain et al., 2020; Mazza et al., 2020; Winkler et al., 2020; Alsharawy et al., 2021; Doshi et al., 2021; Favieri et al., 2021; Orrù et al., 2021; Quadros et al., 2021; Broche-Pérez et al., 2022a,b).

Following other authors (Alon et al., 2020a,b), we agree that increased levels of anxiety and depression, fear of contagion, and worse quality of sleep may be pooled by cultural factors, mainly the domestic management that involved working mothers in the care of children after the closure of nursery schools and childcare centers. We suggest that fear of contagion may be associated with the forethought of the impact of contagion on the management of domestic issues that would increase the stress due to stressful events (Bitan et al., 2020; Uddin, 2021). The literature shows that women over men tend to prefer jobs with a good work-life balance (Burnett et al., 2010; Mellner et al., 2014; Pace and Sciotto, 2021). As a matter of fact, this aspect might have influenced the stress perception due to working from home and to other work aspects that interfered with home management during the pandemic (İlkkaracan and Memiş, 2021; Lonska et al., 2021). Rossi et al. (2020) pointed out that Italian women who responded to a questionnaire on mental health during the lockdown reported higher levels of adjustment disorders, anxiety, depression, and perceived stress than men. Furthermore, according to Cori et al. (2021), women were more likely to consider the consequences of COVID-19 infection more serious than men, with greater compliance with government-imposed rules to contain the spread of the virus.

The lack of significance between those who had an infected loved one and those who did not, is quite unexpected and it shows that the fear of contracting COVID-19 does not depend on the bond of affection. Moreover, the absence of significant results among people who were infected compared to those who were not can be explained by the inhomogeneity of the two groups (low number of the former compared to the latter). Likewise, the absence of statistical significance among people who have lost a loved one compared to their opposite.

Our second research question was about the process of sense- making of the experience during the lockdown and we hypothesized a consistency between the content reported in the open-ended question and the results of the questionnaires measuring stress level, fear of contagion, and well-being. The content analysis points out two main narratives: the description of emotional states (overall hedonic value of the period), experiences, and feelings opposed to the objective description of daily activities and how they managed to reconcile them (study, family). The second concerns the emotional connotation of the experience through a very rich set of adjectives and expressions from positive to negative ones. Descriptions of changes in daily routines or interpersonal relationships due to the first lockdown already emerged in the narrative research study by Venuleo et al. (2022), however, in our study, participants also provided evaluations of the experience related to this change, which are both negative (fear, loneliness, stress, nightmarish, boring, anger, sadness) and positive (serenity, reflection, freedom, opportunity, strength). During the lockdown, participants in our study recounted feelings of fear related to possible contagion as well as loneliness and sadness at being forced to stay locked in their homes (e.g., Marinaci et al., 2021). At the same time, they tried to reconcile their various activities (work, family) as best as they could, even if there were management difficulties. It was both a period in which they could care for intra-family relations and a period of loneliness and sadness for not being able to visit loved ones, even sick ones. The study by Tomaino et al. (2021) similarly highlighted both the presence of negative emotions and coping modes implemented to deal with lockdown measures. Coherently, authors also noted that the main concerns are related to work aspects and dealing with the separation of one’s private and work spheres.

Still, results need to be interpreted in the light of some limitations. The first limitation concerns the convenience sample, which did not allow us to analyze, for example, differences between those who had or had not contracted the virus, or to analyze differences in age or health condition with respect to the lockdown narratives. However, the survey made it possible to collect data from all over Italy and suggested possible territorial differences to be investigated in future studies. Another limitation is the use of self-report measures in the online survey. These measures, especially if administered remotely, may be subject to data collection bias, as they do not ensure consistency of context when compiling the research protocol. As reported in other studies on the COVID-19 pandemic, the adoption of an online survey was the best solution when social distancing measures limited data collection. Finally, although this study is informative regarding how people lived during the first lockdown, inferences drawn from this study through descriptive analyses do not define causal association.

Despite these limitations, from a methodological point of view this study highlights the importance of integrating information from validated quantitative instruments with information gathered in depth by qualitative insight. A mixed-method approach made it possible, on the one hand, to quantitatively understand the impact of COVID-19 on people’s daily lives and on their level of well-being, stress, and fear through the use of validated instruments; on the other hand, to collect and understand in depth how participants experienced the period of the first lockdown on each aspect of their lives through the use of a narrative stimulus (e.g., Parola, 2020; Cipolletta et al., 2022).

In conclusion, our study contributes to enriching scientific knowledge regarding the period of the first lockdown in Italy, assessed at approximately 1 month after the third phase.

A month after the first wave of the pandemic, participants in our study were improving their overall sense of wellbeing. The quantitative analyses tell us that they felt better at the time of the survey (in terms of quality of life, health, sleep, fear of contagion) than they did during the lockdown; they also were able to partially return to normalcy, with the possibility of accessing places of entertainment while maintaining social distances and wearing a face mask. People’s qualitative narratives of that period denote anxiety, sadness, fear of contagion, but also the discovery of a new daily life as an opportunity to experience relationships and activities in a different way (related to work, school, family, and leisure time management, etc.).

Participants experienced a high level of COVID-19-related fears that affected their personal well-being, mediated, however, by the level of perceived stress. Stress level, as we know, is influenced by difficulties, major events, and changes in adaptive resources daily. Probably, the unpredictability or uncontrollability of that moment, as well as the fact of being overburdened by everyday life, also influenced the COVID-19-related fears.

The persistence of the fear and the emotional ambivalence in their narratives suggest that psychologists and social professionals should pay particular attention to the sense-making of an unprecedented event such as the COVID-19 pandemic to support a correct reconstruction of experiences as well as of the emotions in the events of that period. Indeed, more vulnerable individuals were more affected by the lockdown: in our study they were less educated, unmarried, and younger adults, while Hébert et al. (2022) pointed out the sufferance of youth gender and sexual minorities. The analysis of the experiences of the first lockdown also allows us to understand what condition people were in when the second wave arrived in the autumn of 2020, resulting in a new lockdown and worsening of the infections. Overall, the results of our study underscore the need for a customized approach in the analysis of the effects and the narratives of the pandemic, in the long term. Given the proportions of the pandemic phenomenon, the experience of the pandemic period has been in flux over the past 3 years. It would therefore be interesting for future studies to understand how after 3 years the pandemic experience has been understood and signified, also because the virus is now close to becoming endemic.

## Data availability statement

The raw data supporting the conclusions of this article will be made available by the authors, without undue reservation.

## Ethics statement

Ethical review and approval was not required for the study on human participants in accordance with the local legislation and institutional requirements. The patients/participants provided their written informed consent to participate in this study.

## Author contributions

VC, MC, EC, GM, VM, MP, and DC: conceptualization. VC, GM, PC, and EC: formal analysis. VC, GM, PC, and MC: data curation. VC, MC, PC, GM, AM, and MV: writing–original draft preparation. All authors contributed to the article and approved the submitted version.

## Conflict of interest

The authors declare that the research was conducted in the absence of any commercial or financial relationships that could be construed as a potential conflict of interest.

## Publisher’s note

All claims expressed in this article are solely those of the authors and do not necessarily represent those of their affiliated organizations, or those of the publisher, the editors and the reviewers. Any product that may be evaluated in this article, or claim that may be made by its manufacturer, is not guaranteed or endorsed by the publisher.

## References

[ref1] Alon T. Doepke M. Olmstead-Rumsey J. Tertilt M. (2020a). *This Time it’s Different: The Role of Women’s Employment in a Pandemic Recession*. National Bureau of Economic Research Working Paper, No. 27660.

[ref2] Alon T. Doepke M. Olmstead-Rumsey J. Tertilt M. (2020b). *The Impact of the Coronavirus Pandemic on Gender Equality*. Covid Economics Vetted and Real-Time Papers, No. 4, pp. 62–85.

[ref3] Alsharawy A. Spoon R. Smith A. Ball S. (2021). Gender differences in fear and risk perception during the COVID-19 pandemic. Front. Psychol. 12:689467. doi: 10.3389/fpsyg.2021.689467, PMID: 34421741 PMC8375576

[ref4] Anguera M. T. Blanco-Villaseñor A. Jonsson G. K. Losada J. L. Portell M. (2020). Editorial: best practice approaches for mixed methods research in psychological science. Front. Psychol. 11:590131. doi: 10.3389/fpsyg.2020.59013, PMID: 33424707 PMC7793979

[ref5] Banerjee A. Kohli N. (2022). Prevalence of loneliness among young adults during COVID-19 lockdown in India. J. Posit. Sch. Psychol. 6, 7797–7805.

[ref6] Bin Helayel H. Ahmed A. Ahmed S. K. Ahmad A. Khan R. Al-Swailem S. A. (2022). Quarantine-related traumatic stress, views, and experiences during the first wave of coronavirus pandemic: a mixed-methods study among adults in Saudi Arabia. PLoS One 17:e0261967. doi: 10.1371/journal.pone.0261967, PMID: 35025910 PMC8758060

[ref7] Biondi M. Iannitelli A. (2020). CoViD-19 and stress in the pandemic: “sanity is not statistical”. Riv. Psichiatr. 55, 1e–6e. doi: 10.1708/3382.33568 32489189

[ref8] Bitan D. T. Grossman-Giron A. Bloch Y. Mayer Y. Shiffman N. Mendlovic S. (2020). Fear of COVID-19 scale: psychometric characteristics, reliability and validity in the Israeli population. Psychiatry Res. 289:113100. doi: 10.1016/j.psychres.2020.113100, PMID: 32425276 PMC7227556

[ref9] Boursier V. Musetti A. Gioia F. Flayelle M. Billieux J. Schimmenti A. (2021). Is watching TV series an adaptive coping strategy during the COVID-19 pandemic? Insights from an Italian community sample. Front. Psych. 12:599859. doi: 10.3389/fpsyt.2021.599859, PMID: 33967845 PMC8097049

[ref10] Broche-Pérez Y. Fernández-Fleites Z. Fernández-Castillo E. Jiménez-Puig E. Ferrer-Lozano D. M. Vizcaíno-Escobar A. E.. (2022a). Female gender and knowing a person positive for COVID-19 significantly increases fear levels in the Cuban population. Int. J. Ment. Health 51, 102–109. doi: 10.1080/00207411.2021.1952739

[ref11] Broche-Pérez Y. Fernández-Fleites Z. Jiménez-Puig E. Fernández-Castillo E. Rodríguez-Martin B. C. (2022b). Gender and fear of COVID-19 in a Cuban population sample. Int. J. Ment. Heal. Addict. 20, 83–91. doi: 10.1007/s11469-020-00343-8, PMID: 32837428 PMC7292241

[ref12] Brooks S. K. Webster R. K. Smith L. E. Woodland L. Wessely S. Greenberg N.. (2020). The psychological impact of quarantine and how to reduce it: rapid review of the evidence. Lancet 395, 912–920. doi: 10.1016/S0140-6736(20)30460-8, PMID: 32112714 PMC7158942

[ref13] Burnett S. B. Gatrell C. J. Cooper C. L. Sparrow P. (2010). Well-balanced families? A gendered analysis of work-life balance policies and work family practices. Gender Manage. 25, 534–549. doi: 10.1108/17542411011081356

[ref14] Casagrande M. Favieri F. Tambelli R. Forte G. (2020). The enemy who sealed the world: effects quarantine due to the COVID-19 on sleep quality, anxiety, and psychological distress in the Italian population. Sleep Med. 75, 12–20. doi: 10.1016/j.sleep.2020.05.011, PMID: 32853913 PMC7215153

[ref15] Chirico F. Afolabi A. A. Ilesanmi O. S. Nucera G. Ferrari G. Sacco A.. (2021). Prevalence, risk factors and prevention of burnout syndrome among healthcare workers: an umbrella review of systematic reviews and meta-analyses. J. Health Soc. Sci. 6, 465–491. doi: 10.19204/2021/prvl3

[ref16] Cipolletta S. Tomaino S. C. M. Rivest-Beauregard M. Sapkota R. P. Brunet A. Winter D. (2022). Narratives of the worst experiences associated with peritraumatic distress during the COVID-19 pandemic: a mixed method study in the USA and Italy. Eur. J. Psychotraumatol. 13:2129359. doi: 10.1080/20008066.2022.2129359, PMID: 36247840 PMC9559052

[ref17] Cohen S. Kamarck T. Mermelstein R. (1983). A global measure of perceived stress. J. Health Soc. Behav. 24, 385–396. doi: 10.2307/2136404 6668417

[ref18] Cori L. Curzio O. Adorni F. Prinelli F. Noale M. Trevisan C.. (2021). Fear of COVID-19 for individuals and family members: indications from the national cross-sectional study of the epicovid19 web-based survey. Int. J. Environ. Res. Public Health 18:3248. doi: 10.3390/ijerph18063248, PMID: 33801074 PMC8003842

[ref19] Crescenzo P. Chirico F. Ferrari G. Szarpak L. Nucera G. Marciano R.. (2021a). Prevalence and predictors of burnout syndrome among Italian psychologists following the first wave of the COVID-19 pandemic: a cross-sectional study. J. Health Soc. Sci. 6:509. doi: 10.19204/2021/prvl5

[ref20] Crescenzo P. Marciano R. Maiorino A. Denicolo D. D’Ambrosi D. Ferrara I.. (2021b). First COVID-19 wave in Italy: coping strategies for the prevention and prediction of burnout syndrome (BOS) in voluntary psychologists employed in telesupport. Psychol. Hub 38, 31–38. doi: 10.13133/2724-2943/17435

[ref21] D’Ambrosi D. Marciano R. Paolucci A. Crescenzo P. Ferrara I. Maiorino A. (2020). L’impatto psicologico del Covid-19 sulla popolazione: analisi descrittiva delle problematiche psicologiche lockdown correlate progetto: sostegno psicologico #iorestoacasa. J. Psychosoc. Syst. 4, 1–14. doi: 10.23823/jps.v4i2.76

[ref22] Doshi D. Karunakar P. Sukhabogi J. R. Prasanna J. S. Mahajan S. V. (2021). Assessing coronavirus fear in Indian population using the fear of COVID-19 scale. Int. J. Ment. Health Addict. 19, 2383–2391. doi: 10.1007/s11469-020-00332-x, PMID: 32837422 PMC7255701

[ref23] Ettman C. K. Abdalla S. M. Cohen G. H. Sampson L. Vivier P. M. Galea S. (2020). Prevalence of depression symptoms in US adults before and during the COVID-19 pandemic. JAMA Netw. Open 3:e2019686. doi: 10.1001/jamanetworkopen.2020.1, PMID: 32876685 PMC7489837

[ref24] Favieri F. Forte G. Tambelli R. Casagrande M. (2021). The Italians in the time of coronavirus: psychosocial aspects of the unexpected COVID-19 pandemic. Front. Psych. 12:551924. doi: 10.3389/fpsyt.2021.551924, PMID: 33854444 PMC8039140

[ref25] Femia G. Federico I. Ciullo V. Provenzano S. De Luca A. Picano F.. (2020). Gli effetti psicologici della pandemia: strategie di coping e tratti di personalità. Cogn. Clin. 17, 119–135. doi: 10.36131/COGNCL20200202

[ref26] Fortunato L. d’Amati G. Taffurelli M. Tinterri C. Marotti L. Cataliotti L. (2021). Severe impact of Covid-19 pandemic on breast cancer Care in Italy: a Senonetwork National Survey. Clin. Breast Cancer 21, e165–e167. doi: 10.1016/j.clbc.2020.10.012, PMID: 33419687 PMC9760413

[ref27] Fossati A. (2010). Traduzione Italiana Della Scala per lo Stress Percepito [Italian Translation of the Perceived Stress Scale]. Milan, Italy: Università Vita-Salute San Raffaele.

[ref28] Gritti P. (2020). Family Systems in the era of COVID-19: from openness to quarantine. J. Psychosoc. Syst. 4, 1–5. doi: 10.23823/jps.v4i1.64

[ref29] Gritti A. Salvati T. Russo K. Catone G. (2020). COVID-19 pandemic: a note for psychiatrists and psychologists. J. Psychosoc. Syst. 4, 63–77. doi: 10.23823/jps.v4i1.70

[ref30] Grossi E. Groth N. Mosconi P. Cerutti R. Pace F. Compare A.. (2006). Development and validation of the short version of the psychological general well-being index (PGWB-S). Health Qual. Life Outcomes 4:88. doi: 10.1186/1477-7525-4-88, PMID: 17105655 PMC1647268

[ref31] Harris M. L. McLeod A. Titler M. G. (2023). Health experiences of nurses during the COVID-19 pandemic: a mixed methods study. West. J. Nurs. Res. 1939459221148825:019394592211488. doi: 10.1177/01939459221148825, PMID: 36625341 PMC9834626

[ref32] Hébert M. Jean-Thorn A. Malchelosse K. (2022). An exploratory mixed-method descriptive analysis of youth coping during the first wave of the COVID-19 pandemic in Quebec. J. Child Adolesc. Trauma 1–14, 1–14. doi: 10.1007/s40653-022-00505-x, PMID: 36532140 PMC9734407

[ref33] Hossain M. A. Jahid M. I. K. Hossain K. M. A. Walton L. M. Uddin Z. Haque M. O.. (2020). Knowledge, attitudes, and fear of COVID-19 during the rapid rise period in Bangladesh. PLoS One 15:e0239646. doi: 10.1371/journal.pone.0239646, PMID: 32970769 PMC7514023

[ref34] İlkkaracan İ. Memiş E. (2021). Transformations in the gender gaps in paid and unpaid work during the COVID-19 pandemic: findings from Turkey. Fem. Econ. 27, 288–309. doi: 10.1080/13545701.2020.1849764

[ref35] Iorio I. Sommantico M. Parrello S. (2020). Dreaming in the time of COVID-19: a quali-quantitative Italian study. Dreaming 30, 199–215. doi: 10.1037/drm0000142

[ref37] Lancia F. (2004). Strumenti per l’analisi dei testi. Introduzione all’uso di T-LAB. Milano: Franco Angeli.

[ref38] Lanciano T. Graziano G. Curci A. Costadura S. Monaco A. (2020). Risk perceptions and psychological effects during the Italian COVID-19 emergency. Front. Psychol. 11:580053. doi: 10.3389/fpsyg.2020.580053, PMID: 33071920 PMC7533588

[ref39] Lee E. H. (2012). Review of the psychometric evidence of the perceived stress scale. Asian Nurs. Res. 6, 121–127. doi: 10.1016/j.anr.2012.08.004, PMID: 25031113

[ref40] Leleszi-Tróbert A. M. Bagyura M. Széman Z. (2022). Elderly care and burden of family carers during the first wave of COVID-19 pandemic. Orv. Hetil. 163, 1654–1662. doi: 10.1556/650.2022.32596, PMID: 36244008

[ref41] Li Z. Ge J. Yang M. Feng J. Qiao M. Jiang R.. (2020). Vicarious traumatization in the general public, members, and non-members of medical teams aiding in COVID-19 control. Brain Behav. Immun. 88, 916–919. doi: 10.1016/j.bbi.2020.03.007 32169498 PMC7102670

[ref42] Limone P. Toto G. A. (2022). Protocols and strategies to use emergency psychology in the face of an emergency: a systematic review. Acta Psychol. 229:103697. doi: 10.1016/j.actpsy.2022.103697, PMID: 35963114

[ref43] Lonska J. Mietule I. Litavniece L. Arbidane I. Vanadzins I. Matisane L.. (2021). Work–life balance of the employed population during the emergency situation of COVID-19 in Latvia. Front. Psychol. 12:682459. doi: 10.3389/fpsyg.2021.682459, PMID: 34421734 PMC8377232

[ref44] Mari E. Fraschetti A. Lausi G. Pizzo A. Baldi M. Paoli E.. (2020). Forced cohabitation during coronavirus lockdown in Italy: a study on coping, stress and emotions among different family patterns. J. Clin. Med. 9:3906. doi: 10.3390/jcm9123906 33272002 PMC7761111

[ref45] Mariani R. Renzi A. di Trani M. Trabucchi G. Danskin K. Tambelli R. (2020). The impact of coping strategies and perceived family support on depressive and anxious symptomatology during the coronavirus pandemic (COVID-19) lockdown. Front. Psych. 11:587724. doi: 10.3389/fpsyt.2020.587724, PMID: 33281647 PMC7691226

[ref46] Marinaci T. Venuleo C. Gennaro A. Sammut G. (2021). Making sense of the COVID-19 pandemic: a qualitative longitudinal study investigating the first and second wave in Italy. Heliyon 7:e07891. doi: 10.1016/j.heliyon.2021.e07891, PMID: 34493989 PMC8413190

[ref47] Mayer B. Helm S. Barnett M. Arora M. (2022). The impact of workplace safety and customer misbehavior on supermarket workers’ stress and psychological distress during the COVID-19 pandemic. Int. J. Workplace Health Manag. 15, 339–358. doi: 10.1108/IJWHM-03-2021-0074

[ref48] Mazza C. Ricci E. Biondi S. Colasanti M. Ferracuti S. Napoli C.. (2020). A nationwide survey of psychological distress among Italian people during the COVID-19 pandemic: immediate psychological responses and associated factors. Int. J. Environ. Res. Public Health 17:3165. doi: 10.3390/ijerph17093165, PMID: 32370116 PMC7246819

[ref49] McAdams D. P. Josselson R. Lieblich A. (2001). Turns in the Road: Narrative Studies of Lives in Transition. Washington, DC: American Psychological Association.

[ref50] Mellner C. Aronsson G. Kecklund G. (2014). Boundary management preferences, boundary control, and work-life balance among full-time employed professionals in knowledge-intensive, flexible work. Nord. J. Work. Life Stud. 4, 7–17. doi: 10.19154/njwls.v4i4.4705

[ref51] Moccia L. Janiri D. Pepe M. Dattoli L. Molinaro M. De Martin V.. (2020). Affective temperament, attachment style, and the psychological impact of the COVID-19 outbreak: an early report on the Italian general population. Brain Behav. Immun. 87, 75–79. doi: 10.1016/j.bbi.2020.04.048, PMID: 32325098 PMC7169930

[ref52] Mourad F. Mangialavori S. Delle Fave A. (2022). Resilience and experience of the COVID-19 pandemic among Italian university students: a mixed-method study. Int. J. Environ. Res. Public Health 19:11714. doi: 10.3390/ijerph191811714, PMID: 36141988 PMC9517496

[ref53] Orgilés M. Morales A. Delvecchio E. Mazzeschi C. Espada J. P. (2020). Immediate psychological effects of the COVID-19 quarantine in youth from Italy and Spain. Front. Psychol. 11:2986. doi: 10.3389/fpsyg.2020.579038, PMID: 33240167 PMC7677301

[ref54] Orrù G. Bertelloni D. Diolaiuti F. Conversano C. Ciacchini R. Gemignani A. (2021). A psychometric examination of the coronavirus anxiety scale and the fear of coronavirus disease 2019 scale in the Italian population. Front. Psychol. 12:669384. doi: 10.3389/fpsyg.2021.669384, PMID: 34220641 PMC8249697

[ref55] Pace F. Sciotto G. (2021). Gender differences in the relationship between work–life balance, career opportunities and general health perception. Sustainability 14:357. doi: 10.3390/su14010357

[ref56] Parola A. (2020). Novel coronavirus outbreak and career development: a narrative approach into the meaning for Italian university graduates. Front. Psychol. 11:2255. doi: 10.3389/fpsyg.2020.02255, PMID: 33192751 PMC7642812

[ref57] Parrello S. Sommantico M. Lacatena M. Iorio I. (2021). Adolescents’ dreams under Covid-19 isolation. Int. J. Dream Res. 14, 10–20. doi: 10.11588/ijodr.2021.1.73858

[ref58] Pfefferbaum B. North C. S. (2020). Mental health and the Covid-19 pandemic. N. Engl. J. Med. 383, 510–512. doi: 10.1056/NEJMp2008017 32283003

[ref59] Quadros S. Garg S. Ranjan R. Vijayasarathi G. Mamun M. A. (2021). Fear of COVID 19 infection across different cohorts: a scoping review. Front. Psych. 12:708430. doi: 10.3389/fpsyt.2021.708430, PMID: 34557117 PMC8453018

[ref60] Richards H. L. Wormald A. O’Dwyer A. Najt P. Eustace J. O’Connor K.. (2023). Healthcare workers beliefs about COVID-19; a longitudinal, mixed methods analysis. Psychol. Health Med. 28, 110–123. doi: 10.1080/13548506.2022.2032773 35089104

[ref61] Rolland F. (2022). Distress and resilience of Paris-Saclay medical students during the first wave of the COVID-19 pandemic. Ann. Med. Psychol. doi: 10.1016/j.amp.2022.05.004, PMID: 35668954 PMC9159789

[ref62] Rosenberg M. Luetke M. Hensel D. Kianersi S. Fu T. C. Herbenick D. (2021). Depression and loneliness during April 2020 COVID-19 restrictions in the United States, and their associations with frequency of social and sexual connections. Soc. Psychiatry Psychiatr. Epidemiol. 56, 1221–1232. doi: 10.1007/s00127-020-02002-8, PMID: 33386873 PMC7778397

[ref63] Rossi R. Socci V. Talevi D. Mensi S. Niolu C. Pacitti F.. (2020). COVID-19 pandemic and lockdown measures impact on mental health among the general population in Italy. Front. Psych. 11:790. doi: 10.3389/fpsyt.2020.00790, PMID: 32848952 PMC7426501

[ref64] Saladino V. Algeri D. Auriemma V. (2020). The psychological and social impact of Covid-19: new perspectives of well-being. Front. Psychol. 11:577684. doi: 10.3389/fpsyg.2020.577684, PMID: 33132986 PMC7561673

[ref65] Saladino V. Auriemma V. Campinoti V. (2022). Healthcare professionals, post-traumatic stress disorder, and COVID-19: a review of the literature. Front. Psych. 12:795221. doi: 10.3389/fpsyt.2021.795221, PMID: 35126205 PMC8813735

[ref66] Schimmenti A. Starcevic V. Giardina A. Khazaal Y. Billieux J. (2020). Multidimensional assessment of COVID-19-related fears (MAC-RF): a theory-based instrument for the assessment of clinically relevant fears during pandemics. Front. Psych. 11:748. doi: 10.3389/fpsyt.2020.00748, PMID: 32848926 PMC7411221

[ref67] Simione L. Gnagnarella C. (2020). Differences between health workers and general population in risk perception, behaviors, and psychological distress related to COVID-19 spread in Italy. Front. Psychol. 11:2166. doi: 10.3389/fpsyg.2020.02166, PMID: 33013555 PMC7499804

[ref68] Sommantico M. Iorio I. Lacatena M. Parrello S. (2021). Dreaming during the COVID-19 lockdown: a comparison of Italian adolescents and adults. Res. Psychother. (Milano) 24:536. doi: 10.4081/ripppo.2021.536, PMID: 34568105 PMC8451218

[ref69] Svantesson M. Durnell L. Hammarström E. Jarl G. Sandman L. (2022). Moral and exhausting distress working in the frontline of COVID-19: a Swedish survey during the first wave in four healthcare settings. BMJ Open 12:e055726. doi: 10.1136/bmjopen-2021-055726, PMID: 35851022 PMC9296999

[ref70] Tarchi L. Crescenzo P. Talamonti K. (2022). Prevalence and predictors of mental distress among Italian red cross auxiliary corps: a cross-sectional evaluation after deployment in anti-COVID-19 operations. Mil. Psychol. 1-14, 1–14. doi: 10.1080/08995605.2022.2069983 PMC10453978 37615558

[ref71] Tomaino S. C. M. Cipolletta S. Kostova Z. Todorova I. (2021). Stories of life during the first wave of the COVID-19 pandemic in Italy: a qualitative study. Int. J. Environ. Res. Public Health 18:7630. doi: 10.3390/ijerph18147630, PMID: 34300081 PMC8304996

[ref72] Uddin M. (2021). Addressing work-life balance challenges of working women during COVID-19 in Bangladesh. Int. Soc. Sci. J. 71, 7–20. doi: 10.1111/issj.12267, PMID: 34230685 PMC8251227

[ref73] Venuleo C. Rollo S. Ferrante L. Marino C. Schimmenti A. (2022). Being online in the time of COVID-19: narratives from a sample of young adults and the relationship with well-being. Mediterranean. J. Clin. Psychol. 10:3236. doi: 10.13129/2282-1619/mjcp-3236

[ref74] Wandschneider L. Batram-Zantvoort S. Alaze A. Niehues V. Spallek J. Razum O.. (2022). Self-reported mental well-being of mothers with young children during the first wave of the COVID-19 pandemic in Germany: a mixed-methods study. Women’s Health (Lond. Engl.) 18:17455057221114274. doi: 10.1177/17455057221114274, PMID: 35997231 PMC9424892

[ref75] Welling M. S. Abawi O. van den Eynde E. van Rossum E. F. C. Halberstadt J. Brandsma A. E.. (2022). Impact of the COVID-19 pandemic and related lockdown measures on lifestyle behaviors and well-being in children and adolescents with severe obesity. Obes. Facts 15, 186–196. doi: 10.1159/000520718, PMID: 34743080 PMC8805051

[ref76] Winkler P. Formanek T. Mlada K. Kagstrom A. Mohrova Z. Mohr P.. (2020). Increase in prevalence of current mental disorders in the context of COVID-19: analysis of repeated nationwide cross-sectional surveys. Epidemiol. Psychiatr. Sci. 29:e173. doi: 10.1017/S2045796020000888, PMID: 32988427 PMC7573458

[ref77] Yao H. Chen J. H. Xu Y. F. (2020). Patients with mental health disorders in the COVID-19 epidemic. Lancet Psychiatry 7:e21. doi: 10.1016/S2215-0366(20)30090-0, PMID: 32199510 PMC7269717

[ref78] Yu H. Li M. Li Z. Xiang W. Yuan Y. Liu Y.. (2020). Coping style, social support and psychological distress in the general Chinese population in the early stages of the COVID-19 epidemic. BMC Psychiatry 20:426. doi: 10.1186/s12888-020-02826-3, PMID: 32854656 PMC7450895

